# HLA Haplotyping from RNA-seq Data Using Hierarchical Read Weighting

**DOI:** 10.1371/journal.pone.0067885

**Published:** 2013-06-28

**Authors:** Hyunsung John Kim, Nader Pourmand

**Affiliations:** Biomolecular Engineering Department, Baskin School of Engineering, University of California, Santa Cruz, Santa Cruz, California, United States of America; Centro Cardiologico Monzino IRCCS, Italy

## Abstract

Correctly matching the HLA haplotypes of donor and recipient is essential to the success of allogenic hematopoietic stem cell transplantation. Current HLA typing methods rely on targeted testing of recognized antigens or sequences. Despite advances in Next Generation Sequencing, general high throughput transcriptome sequencing is currently underutilized for HLA haplotyping due to the central difficulty in aligning sequences within this highly variable region. Here we present the method, HLAforest, that can accurately predict HLA haplotype by hierarchically weighting reads and using an iterative, greedy, top down pruning technique. HLAforest correctly predicts >99% of allele group level (2 digit) haplotypes and 93% of peptide-level (4 digit) haplotypes of the most diverse HLA genes in simulations with read lengths and error rates modeling currently available sequencing technology. The method is very robust to sequencing error and can predict 99% of allele-group level haplotypes with substitution rates as high as 8.8%. When applied to data generated from a trio of cell lines, HLAforest corroborated PCR-based HLA haplotyping methods and accurately predicted 16/18 (89%) major class I genes for a daughter–father-mother trio at the peptide level. Major class II genes were predicted with 100% concordance between the daughter–father-mother trio. In fifty HapMap samples with paired end reads just 37 nucleotides long, HLAforest predicted 96.5% of allele group level HLA haplotypes correctly and 83% of peptide level haplotypes correctly. In sixteen RNAseq samples with limited coverage across HLA genes, HLAforest predicted 97.7% of allele group level haplotypes and 85% of peptide level haplotypes correctly.

## Introduction

Hematopoietic stem cell transplantation (HSCT) has successfully treated a wide variety of diseases including autoimmune disorders, rare genetic diseases and blood cancers [1]. Success of allogenic HSCT treatments is highly correlated with matching of Human Leukocyte Antigen (HLA) alleles between donor and recipient [2]. The process of matching a donor and recipient requires HLA haplotyping, a process that typically requires specialized antibody or targeted DNA based tests [3]. The utility of high throughput sequencing methods has been restricted by the need for specialized primer sets to enrich targeted DNA or RNA sequences [4–10]. The use of untargeted RNA-seq data for HLA haplotyping has not seen much development despite dramatic reductions in sequencing costs. This is unfortunate, as RNA-seq assays have proven to be useful tools in personalized medicine for the subclassification of cancers including breast, prostate, leukemias and lymphomas [11–15]. In cases where a patient would benefit from sequencing of RNA and whose disease would require HSCT, it would be cost effective to predict HLA haplotypes directly from RNA-seq data rather than performing an additional specialized test to determine HLA haplotype.

Predicting HLA haplotypes, however, has been historically a difficult task [16]. This is due to the fact that the HLA genes reside in the most polymorphic region of the human genome, the Major Histocompatibility Complex (MHC) [17]. As a result of balancing selection, the number of known haplotypes for many HLA genes is in the thousands [18,19]. Despite the diversity of the region, a high degree of sequence similarity exists between known haplotypes. The unique assignment of a short read to an allele is nearly impossible due to sequence similarity between alleles. The hierarchical nature of the haplotypes (see Methods for details) and the sampling bias, with over representation of most common haplotypes in public databases (e.g. IMGT), further add to the complexity. For these reasons, traditional techniques for assigning mapping qualities or confidence in alignments cannot be applied for this region [20].

Despite the difficulty of predicting HLA haplotypes, attempts to call HLA haplotypes from short read data are numerous. Earlier studies utilized targeted resequencing and the generous read length of the Roche 454 to predict haplotypes [4–10]. Recent studies employ targeted resequencing in conjunction with Illumina sequencers to generate haplotypes [21]. However, at the time of this publication only two publicly available haplotyping tools, HLAMiner and seq2HLA, are available for the HLA haplotyping directly from RNA-seq reads [22,23]. HLAMiner utilizes a targeted *de novo* assembly technique to rebuild the genes and then check the haplotype. While the method effectively handles sequencing errors and can potentially increase phasing information, it is limited by the assembly technique that relies on overlapping kmers just 15 nucleotides long. The second available tool, seq2HLA utilizes an alignment-based method to predict low resolution haplotypes, but is unable to provide predictions at higher resolutions.

Here, we present HLAforest, an alignment-based method that exploits the implicit hierarchy of HLA nomenclature to weight alignments systematically and procedurally predict haplotypes individually. This approach addresses the combinatorial problem of selecting paternal and maternal alleles prior to scoring the most likely haplotype pairs. Additionally, by aligning the entire read to the reference, HLAforest maximizes the information contained within paired reads. Predictions from HLAforest are more accurate at clinically relevant HLA resolutions, due to the topology of the trees generated. Finally, this method exploits the efficiency of modern short read alignment tools and is easily scaled with parallelized computing.

## Methods

### HLA Nomenclature

For both historical and functional reasons, HLA haplotypes are defined hierarchically [24,25]. HLA molecules are divided into two classes: Class I molecules are expressed on almost all nucleated cells in the human body and present self as well as foreign peptides [26]. Class II molecules present foreign peptides and are only expressed by specialized antigen-presenting cells such as macrophages, B-lymphocytes and dendritic cells. Both classes of molecules are responsible for peptide-presentation to T-cells for antigen recognition. The peptide-binding domains of class I molecules are encoded by a single gene, whereas the peptide-binding domains of class II molecules are encoded by two genes.

The first hierarchical unit of HLA genes is the genes themselves. There are three major class I molecules (*HLA-A, HLA-B, HLA-C*), three minor class I molecules (*HLA-E, HLA-F, HLA-G*), three major class II molecules (*HLA-DP, HLA-DQ, HLA-DR*) and two minor class II molecules (*HLA-DM, HLA-DO*). Because class II molecules consist of two genes, haplotypes are further subdivided into an ‘A’ and ‘B’ locus, eg *HLA-DRA* and *HLA-DRB*. The IMGT database also contains the non-classical molecules *MicA*, *MicB*, *TapA*, and *TapB* that do not participate in antigen presentation. However, TapA and TapB are important for the transportation and loading of antigenic peptides onto classical HLA molecules.

Each HLA gene is subdivided into up to four subclassifications: allele group, peptide, nucleotide and intron. Allele groups originated from early serotyping experiments and represent sets of peptide sequences with high levels of sequence homology. The peptide level represents unique amino acid sequences. The nucleotide level represents unique nucleotide sequences that generate the same peptide sequence. The intron level represents polymorphisms that occur in non-coding portions of the gene, such as introns. These subclassifications are denoted by a 2 digit number separated by colons, e.g., the haplotype HLA-A*01*: 02:03:04* is a haplotype for gene ‘*A*’ with the allele group ‘*01*’, peptide sequence of ‘*02*’, nucleotide sequence of ‘*03*’ and intronic sequence of ‘*04*’. A haplotype of HLA-A*01: 02:01:01 belongs to the same peptide group as HLA-A*01: 02:03:04, but has a unique nucleotide sequence.

The resolution of HLA haplotyping assays is typically described by the number of significant digits the test can discriminate. For example, a 2 digit assay will reveal the allele group; whereas, a 4 digit assay will reveal the unique peptide sequence. 2 digit assays are typically referred to as “low resolution typing”; whereas, 4 digit assays are referred to as “high resolution typing”.

### Alignment

Version 3.10.0 of the IMGT HLA nucleotide database was downloaded as a FASTA file and was used as a reference for Bow tie alignments. Null alleles were excluded from the set of known alleles, as intronic reads can artificially inflate the scores of those alleles. Reads mapping to any HLA haplotype in the IMGT were filtered using Bow tie v-0.12.8 using default options [27]. Reads that aligned at least once to any IMGT reference HLA haplotype were kept using Bow tie’s option to write aligned reads to a new file. Filtered reads were then realigned to the IMGT database, this time reporting all possible alignments. Alignments with exact matches excluded any alignment with > 0 mismatches. For read lengths greater than 100bp, the maximum insert size was set to the sum of simulated fragment length and three standard deviations of fragment size. All other parameters were set to bow tie defaults.

### Alignment Tree Building

**Figure 1:**
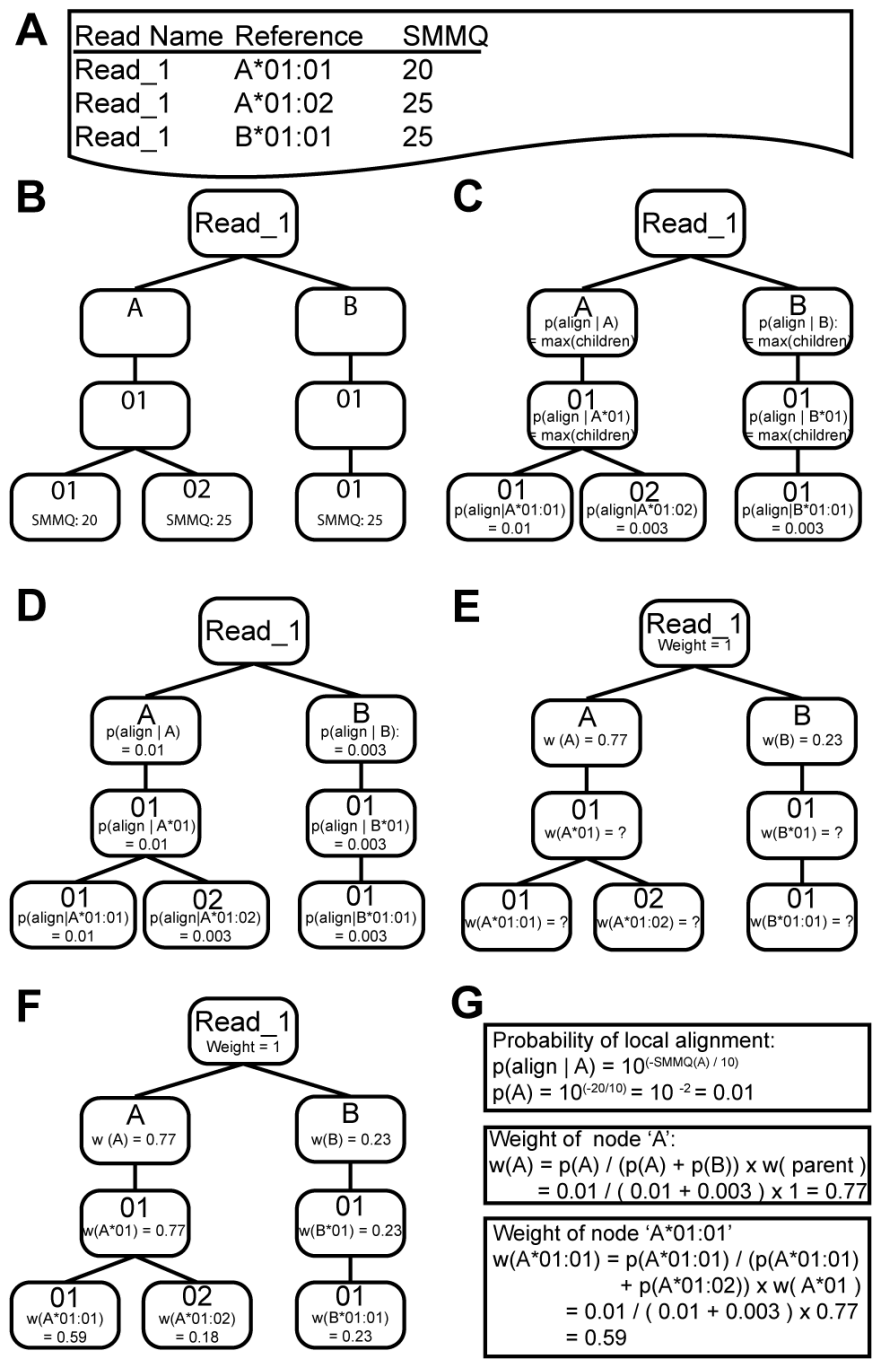
Method for building a weighted read tree. Given a set of alignments (a) for a single read, a tree is built such that all possible alignments are leaf nodes (b). Gene, allele group, peptide, nucleotide and intronic digits are represented as nodes on the tree. Sum of mismatch qualities (SMMQs) are converted to alignment probabilities for leaf nodes (c). Probabilities are then distributed upwards such that the probability of a parent node is equal to the maximum probability of its children (d). Weights are distributed downwards in such that the weight of a node is dependent on the local probability of the node and the weight of the parent child (e & f). Equations used for generating probability of an alignment and weights of example nodes are outlined (g).

A tree is built for each read given the set of possible alignments generated during the mapping step (Figure 1). This tree describes the entire set of alignments such that each leaf in the tree represents an alignment of a read to a specific HLA allele (Figure 1b). The root of the tree is an empty node with no biological significance. The descendants of the root node represent a specific HLA gene (e.g., *HLA-A, HLA-B*, and *HLA-C* are each represented as unique nodes under the root node). The structure of each gene subtree is defined by the HLA nomenclature where the second level represents the allele group, the third level represents unique peptide sequence, the fourth level represents synonymous nucleotide sequences and the fifth level represents differences in non-coding regions. For example, direct descendants of root nodes correspond to genes. The descendants of gene nodes correspond to allele groups and this pattern continues until a leaf is reached.

### Generating Weights for Each Node in a Read Tree

**Figure 2:**
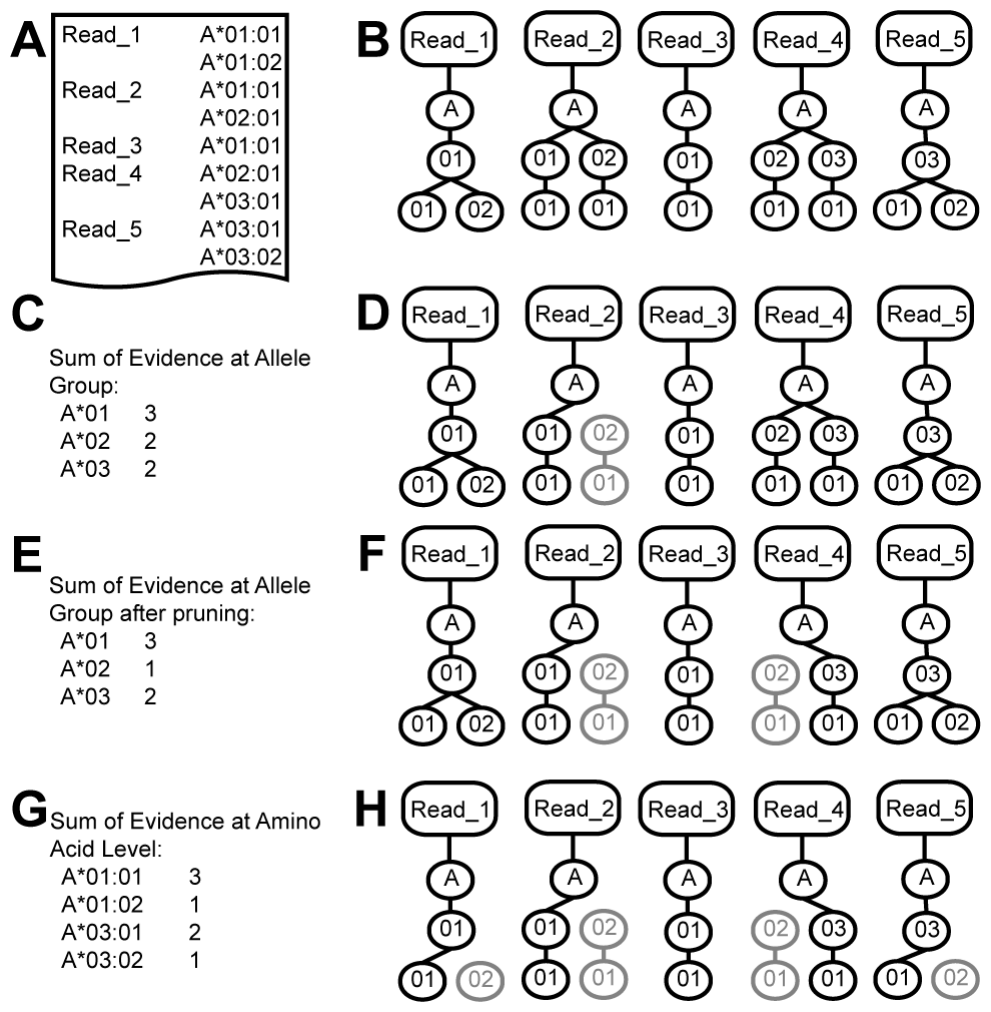
An example of the top-down pruning algorithm. Given a set of reads and their alignments (a), read trees are built for each read (b). The evidence for each allele group is determined by taking the sum of the evidence of all allele groups represented in the trees (c). Here it is assumed that the weight of each node is 1. The allele group with the maximum evidence is assumed to be the primary allele group for each gene and edges in trees containing the primary allele group are pruned (d). After pruning, the trees are reweighted and the evidence for each allele group (e). The second highest scoring allele group is then considered to be the minor haplotype. Read trees are then repruned such that only edges supporting the primary or secondary allele group remain (f). The process repeats itself iteratively until the most likely leaf nodes remain (g and h).

An alignment tree is built for each read given the set of possible alignments generated during the mapping step. Local alignment probabilities are calculated for each leaf node by first identifying mismatched positions in the alignment between the read and a reference allele (Figure 2b). The phred quality scores of mismatched positions are summed and the sum is exponentiated in order to generate a probability (Figure 2c). More specifically:

$$p_{leaf}=10^{-\left(\sum_{i}\frac{q_{i}}{10}\right)}$$

where *p*_*leaf*_ represents the local alignment probability of a leaf and *q* represents to a phred quality value corresponding to a mismatched base at position *i* in the read. These probabilities are then distributed upwards through the tree by assigning the maximum probability of all children to the current node (Figure 2e),

$$p_{node}=\max\left(p_{children}\right)$$

Once probabilities have been distributed throughout the tree, they are converted to weights. The weighting procedure distributes the total evidence provided by the read to all nodes of the tree, such that

$$w_{node}=w_{parent}\times \frac{p_{node}}{\sum p_{siblings}}$$

In the above equation, *w* is the weight of a node and *p* is the local probability of a node (Figure 2f and g). Each node can only have as much weight as its parent, and all weight contained in a node is distributed amongst its children. In this study, all root nodes were given a weight of 1; this weight, however, may be modified to reflect variable read lengths.

### Haplotype Prediction

Haplotypes are predicted using a greedy, top down, iterative pruning algorithm (Figure 2). The algorithm begins by attempting to select a primary and secondary haplotype for each HLA gene at the allele group level. Here a primary haplotype is defined as the haplotype with greater representation in an RNA-seq library. A secondary haplotype is the haplotype with less sequence representation. Selecting a primary haplotype begins by summing the weighted evidence of all read trees at the allele group level (Figure 2b and c). For each gene in the IMGT reference database, the allele group with the highest score is chosen as the primary haplotype (Figure 2c).

After primary haplotypes are chosen, a temporary set of pruned trees is generated to predict the secondary haplotype. Trees that contain evidence for any of the primary haplotypes are pruned by removing edges that do not support primary haplotypes. Secondary haplotypes are predicted by summing the weighted evidence of pruned trees and selecting the haplotype with the second highest score. Read trees are then repruned such that only edges that belong to the primary or secondary allele group remain. In the event that a read tree supports both primary and secondary haplotypes, it is assigned to the haplotype with greater weight. If the weights between the primary and secondary haplotypes are equal, the read is randomly assigned to a haplotype. Nodes of pruned trees are then reweighted using the local alignment probabilities generated in the tree-building step.

Following the selection of primary and secondary haplotypes at the allele group level, protein level haplotypes are chosen using the same methodology used to predict primary haplotypes. The sum of node weights over all pruned trees are generated at the protein level. The protein level haplotype with the maximum score is chosen for both the primary and secondary haplotypes. Trees are then repruned to reflect the protein level haplotypes and the process iterates until the nucleotide and intron level haplotypes are similarly chosen.

In some instances, an individual may have a primary and secondary haplotype that are the same at the allele group level, but differ at the protein or nucleotide level. In these instances, the secondary haplotype is only assigned if the sum of its evidence exceeds 5% of the primary haplotype. This percentage was determined empirically (data not shown).

### Simulations

Simulations were generated to test the effect of read length, sequencing depth and error rate on the accuracy of this HLA haplotyping method. Each of these variables was tested independently. Two haplotypes were randomly selected for a subset of genes represented in the IMGT reference database. In this case, *HLA-A*, *HLA-B*, *HLA-C* and *HLA-DRB1* were chosen as these genes represent the majority of diversity in the IMGT database (Table S1 and Figure S1).

Reads were simulated using ART [28]. ART uses empirically derived models to generate simulated reads with a realistic distribution of errors with regards to position. Simulations tested the effect of substitution rate, coverage, and read length on the accuracy of the method. Paired read lengths of 37 nt, 50 nt, 75 nt, 100 nt and 200 nt were tested. Coverage of 1x, 10x, 25x, 50x, 100x and 500x for each selected gene. Varying substitution rates were generated by modifying the quality shift parameter of the ART simulator. Higher quality shift values resulted in reads with fewer substitutions. The substitution rate for each quality shift value was calculated by dividing the total number of substituted bases by the total number of simulated bases. For 100nt reads, quality shift values of 0, 3, 6, 9, 12, and 30 resulted in substitution rates of 8.8%, 4.4%, 2.2%, 1.1%, 0.5% and .009%, respectively. In addition, simulations using all genes present in the IMGT were also performed. 5,000 simulations were performed for each variable. Paired reads were used in all simulations. Insertion and deletions were not included in simulation. Exact parameters used for simulation are described in Table S2.

### Calculating Simulation Accuracy

Predictions were considered to be accurate if the true haplotype (determined by selection during simulation or through external validation) was consistent at the allele group, peptide, nucleotide or intron level. Accuracy was assessed over all levels present in the true haplotype. If the true allele was only typed to the peptide level, accuracy was not assessed at the nucleotide or intron level. For example, the reference allele HLA-A*02: 90 is only typed to the peptide level and accuracy at the nucleotide level cannot be determined for this allele. Of the 8,631 sequences present in the IMGT database: 87 (1%) are typed to the allele group level; 6,014 (70%) are typed to the peptide level; 2,308 (27%) are typed to the nucleotide level; and 222 (2%) are typed to the intron level.

### Cell Line Trio Data

Cell line Trió RNA-seq data was downloaded from the UCSC Encode release 4 (August 2012). Raw fastq files for GM12878 rep2v2 were downloaded from the UCSC genome browser. Raw fastq files for gm12891 (rep2v2) and gm12892 (rep3v2) were also downloaded from the UCSC genome browser. The number of reads available for each dataset are presented in Table S3.

### HapMap Data

Fifty RNAseq samples were sequenced in a study by de Bakker et al and HLA haplotypes were subsequently reported by Montgomery et al [29,30]. RNA-seq samples were obtained from the NCBI SRA with the study accession id ERP000101. The accession numbers of the individual samples presented in this study are available in Table S4.

### Colorectal Cancer Data

Warren et al tests HLAMiner on sixteen colorectal cancer RNAseq datasets. These samples were HLA Haplotyped by Sanger Sequencing, for which 87 out of 96 possible Class I haplotypes were reported [22]. Corresponding RNAseq data with the NCBI SRA study accession id SRP10181 was downloaded and used as input for HLAforest. Sample accession numbers are available in Table S5.

### Implementation

HLAforest is implemented in perl and is free for academic use under the Apache License. It can be downloaded (http://code.google.com/p/hlaforest). HLAforest uses bioperl to read in FASTA files. Alignments use Bow tie, although any alignment tool can be used to generate SAM alignments for use as input to HLAforest.

## Results

### Simulation Results

**Figure 3:**
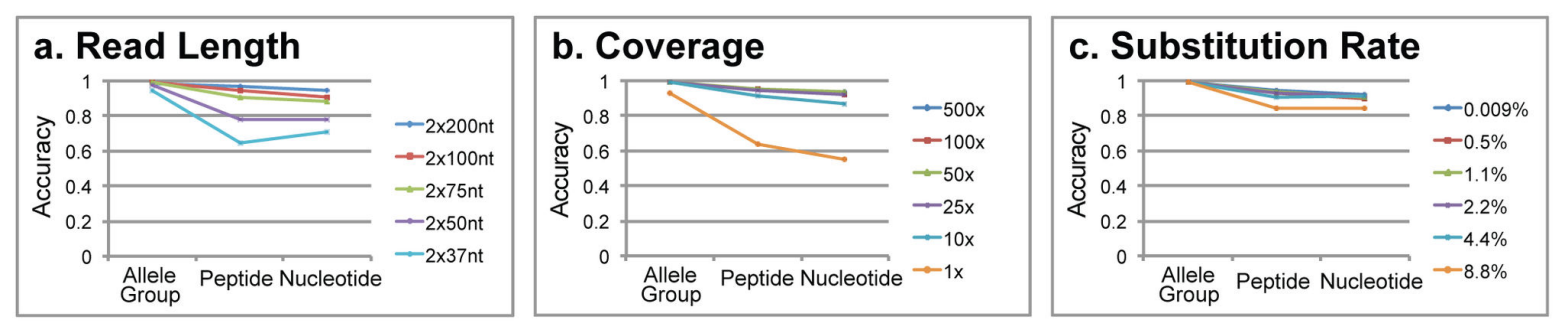
Simulation results showing the effect of read length (a), sequencing depth (b), and substitution rate (c) on average accuracy of HLA-A, HLA-B, HLA-C, and HLA-DRB1.

Simulations were performed to test the effect of read length, coverage depth and substitution rate on the accuracy of the method. These simulations were performed only on the HLA genes *HLA-A*, *HLA-B*, *HLA-C*, and *HLA-DRB1* as these represented the majority of diversity present in the IMGT database (Figure S1). The average of prediction rates over these four genes is reported (Figure 3). Performance of each individual gene is available in Table S2. Simulations were also performed using all genes in the IMGT database in order to test the accuracy on each individual gene. HLAforest performed very well at the allele group level, achieving > 99% accuracy in all situations except for very low coverage (1x) and short read lengths (2x37nt and 2x50nt). Peptide level accuracy varied more throughout the simulations with shorter read lengths contributing most to a decline in accuracy. HLAforest performed well at varying levels of coverage and with substantial substitution rates.

### Simulated Effect of Read Length

Accuracy was largely affected by read length, with longer reads providing substantially better results. Read lengths of 2x50, 2x75, 2x100, and 2x200 nt were tested for their effect on accuracy (Figure 3a). Greater read lengths provided more accurate results, with 2x200 nt achieving very high accuracy (96.7%) at the peptide level. At 2x100 nt an average accuracy of 94% was achieved. At lower read lengths, accuracy declined at the peptide level but increased at the nucleotide level. This effect is artifactual as accuracy is not assessed at the nucleotide level if the true haplotype is only typed to the peptide level. Allele group level suffered with 2x37nt and 2x50nt reads, achieving 94.5% and 97.6% accuracy, respectively. However, accuracy was above 98.7% for 2x75nt, 2x100nt and 2x200nt read lengths.

### Simulated Effect of Sequencing Depth

Simulations showed a minimal effect of increasing sequencing depth on accuracy. Total coverage amounts of 1x, 10x, 25x, 50x, 100x and 500x for each chosen haplotype were tested (Figure 3b). Although high resolution performance suffered at very low coverage (64% at 1x), accuracy jumped to 92% with just 10x coverage. Increasing coverage above 25x had minimal effect on high resolution accuracy. Peptide level accuracy was above 94.9% for all coverage levels above 25x. Allele group level accuracy was above 98.9% at coverage levels greater than 10x.

### Simulated Effect of Substitution Rate

Substitution rates of 0.009%, 0.5%, 1.1%, 2.2%, 4.4%, and 8.8% were assayed in order to test the effect of sequencing error on the accuracy of the method. Substitution rate had minor effects on the accuracy of the method. With substitution rates below 2.2%, peptide level accuracy was above 93%. At 4.4%, accuracy declined to 90%. With a large substitution rate of 8.8%, peptide level accuracy was still respectable at 84%. Allele group level accuracy was above 98.9% for all substitution rates tested.

### Simulations Using All Genes

Simulations using all genes in the IMGT database rather than just *HLA-A*, *HLA-B*, *HLA-C* and *HLA-DRB1* were conducted to see the accuracy of the method on each individual gene (Table S2). Low resolution accuracy was above 98% for most genes except for a few selected genes (DPA1, DPB1, DRB6, MicA, MicB, Tap1, Tap2, V). Peptide level accuracy was above 91% for HLA-A, HLA-B, HLA-C, but fell below 90% for remaining genes.

### Simulations Allowing No Mismatches during Alignment

Disallowing mismatches during the alignment step resulted in higher accuracy when substitution rates were sufficiently low (Table S2). At 0.009% error, peptide level accuracy was 94.3% when mismatches were allowed, but increased to 95.6% when only exact matches were utilized. However, at higher substitution rates, performance declined achieving an accuracy of 55% at 1.1% error rate. At 0.009% substitution rate, accuracy was improved by 1-2% over all conditions tested when no mismatches were allowed during alignment. With a low substitution rate and sufficiently high coverage, it may be beneficial to restrict the number of mismatches during alignment. Boegel et al. reports a similar effect and recommends allowing only a single mismatch during alignment [23].

### Read Weighting and Tree Pruning Reduces Noise

**Figure 4:**
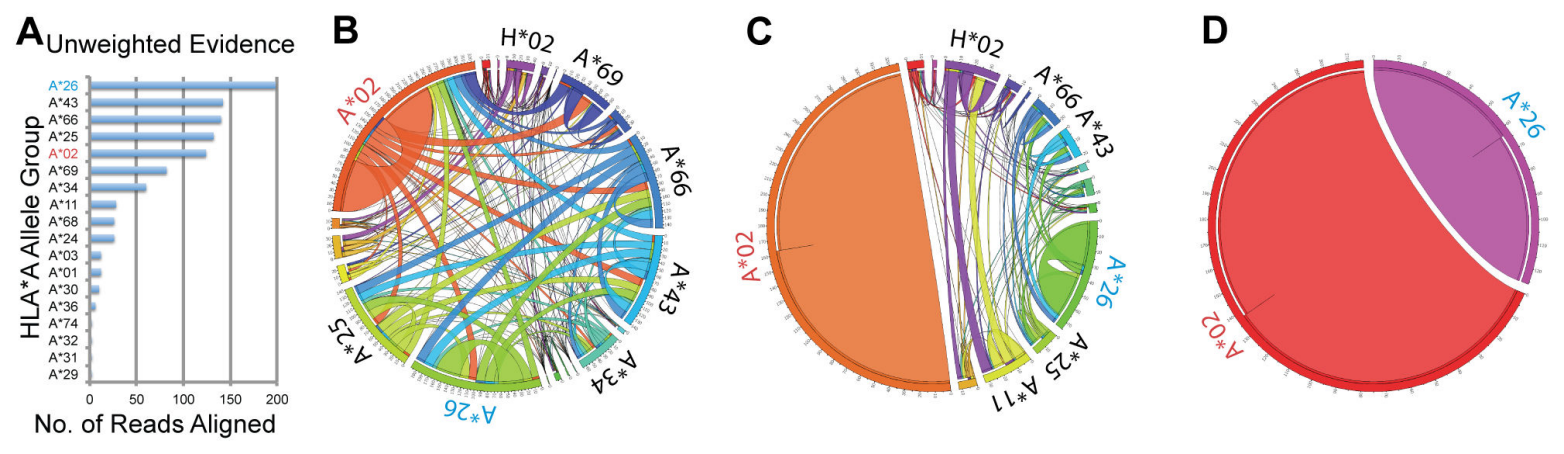
Effect of read weighting and tree pruning on predicting allele group. In this simulated example, HLA-A*02: 90 (labeled in red text) and HLA-A*26: 30 (labeled in blue text) are the true alleles. (a) The maximum number of reads mapping to any descendant of allele groups are shown. These results represent a naive attempt at predicting haplotypes from RNA-seq data where reads are unweighted. (b) Evidence for each allele group after building weighted read trees. Allele groups are labeled on the outermost circle. Arcs connecting allele groups have widths proportional to the amount of evidence that shared between connected allele groups. Here it is already evident that A*02 and A*26 have the most evidence, but other haplotypes have substantial evidence. (c) The effect of pruning read trees after selecting the primary haplotype (A*02) clearly distinguishes the secondary allele group (A*26) from other allele groups. (d) A final pruning step removes all ambiguous edges and assigns all evidence to the true allele groups.

An example demonstrating the effect of read weighting and tree pruning can be visualized in Figure 4. Here, one hundred 2x100 nt reads were sampled for the HLA haplotypes HLA-A*02*: 90* and HLA-A*26: 36. The difficulty of predicting HLA allele groups without read weighting or tree pruning can be seen in Figure 4a, which charts the maximum number of reads that map to a child member of an allele group. Although HLA-A*26 is ranked first in the number of reads supporting that allele, three incorrect allele groups rank above the true allele group of HLA-A*02.

Hierarchically weighting and pruning the simulated reads allows for the reduction of noise and for accurate prediction of allele groups in this example. The hierarchical weighting procedure provides multiple benefits. First it distributes all the evidence a single read could provide to all possible alignments, rather than giving equal weight to all alignments. Secondly, it allows for the visualization of the amount of shared evidence between all allele groups (Figure 4b). After weighting has been applied, HLA-A*02 and HLA-A*26 contain the majority of evidence, however much of the evidence for the incorrect allele groups remain. The intermediate pruning step, where ambiguous evidence is assigned to the primary allele group, significantly reduces noise. Figure 4c shows that post pruning, the secondary allele group with the most evidence is clearly A*26. The final pruning step removes all ambiguous evidence (Figure 4d).

### Cell Line Trio

Three cell lines, gm12878, gm12891, and gm12892, representing a daughter–father-mother trio, respectively, were used to test this method. It is expected that the daughter (gm12878) would carry a set of alleles from each parent. 2x75bp reads were used to predict haplotypes for all genes present in the IMGT database (Table S3).

When compared to haplotypes determined by targeted resequencing and Sanger sequencing as performed by Erlich et al. [5], all HLA class I alleles were recapitulated for gm12878 at the peptide level (Table 1). Most class I alleles were recapitulated for gm12891 and gm12892, except for those that were found to be discordant when compared to gm12878. In all, sixteen out of eighteen (89%) of class I molecules were called consistently with previous studies. Accuracy of other genes, assessed by looking for consistent predictions between the daughter (gm12878) and her parents, had accuracy similar to major class I genes.

**Table 1:** Predicted haplotypes of major HLA genes on the daughter-father-mother trio of cell lines using exact alignments.

**Gene**	**Father (gm12891)**		**Mother (gm12892)**		**Daughter (gm12878)**	
	**Primary**	**Secondary**	**Primary**	**Secondary**	**Paternal**	**Maternal**
**A**	01:01:01**	24:02:01**	11:01:18** ^§^	02:01:01**	01:01:01**	11:01:01** ^§^
**B**	08:01:01**	07:02:01**	15:01:01**	15:01:20^†^	08:01:01**	56:01:01** ^†^
**C**	07:02:01**	07:02:01* ^‡^	01:02:01**	04:01:01**	07:01:01** ^‡^	01:02:01**
**DPA1**	01:03:01	01:03:01	02:01:01	01:03:01	01:03:01	02:01:01
**DPB1**	04:01:01	03:01:01	14:01	06:01	04:01:01	14:01
**DQA1**	05:01:01	01:02:01	01:01:01	01:01:01	05:01:01	01:01:01
**DQB1**	02:01:01	06:02:01	05:01:01	05:01:01	02:01:01	05:01:01
**DRA**	01:02:03^§^	01:02:02	01:01:01	01:01:01	01:02:02^§^	01:01:01
**DRB1**	03:01:01	15:01:01	01:01:01	01:01:01	03:01:01	01:01:01

* Consistent at allele group level with haplotypes from Erlich, et al. [5]

** Consistent at peptide level with haplotypes from Erlich, et al.

† Inconsistent between parent and daughter at allele group level

‡ Inconsistent between parent and daughter at peptide level

§ Inconsistent between parent and daughter at nucleotide level

Accuracy of haplotyping was assessed over the fifteen genes with sufficient coverage (greater than 1% of all mapped reads supporting the gene). Of the thirty alleles in these genes, twenty-six were predicted consistently at the peptide level, two were predicted consistently at the allele group level and two were completely miscalled (Table S3).

**Figure 5:**
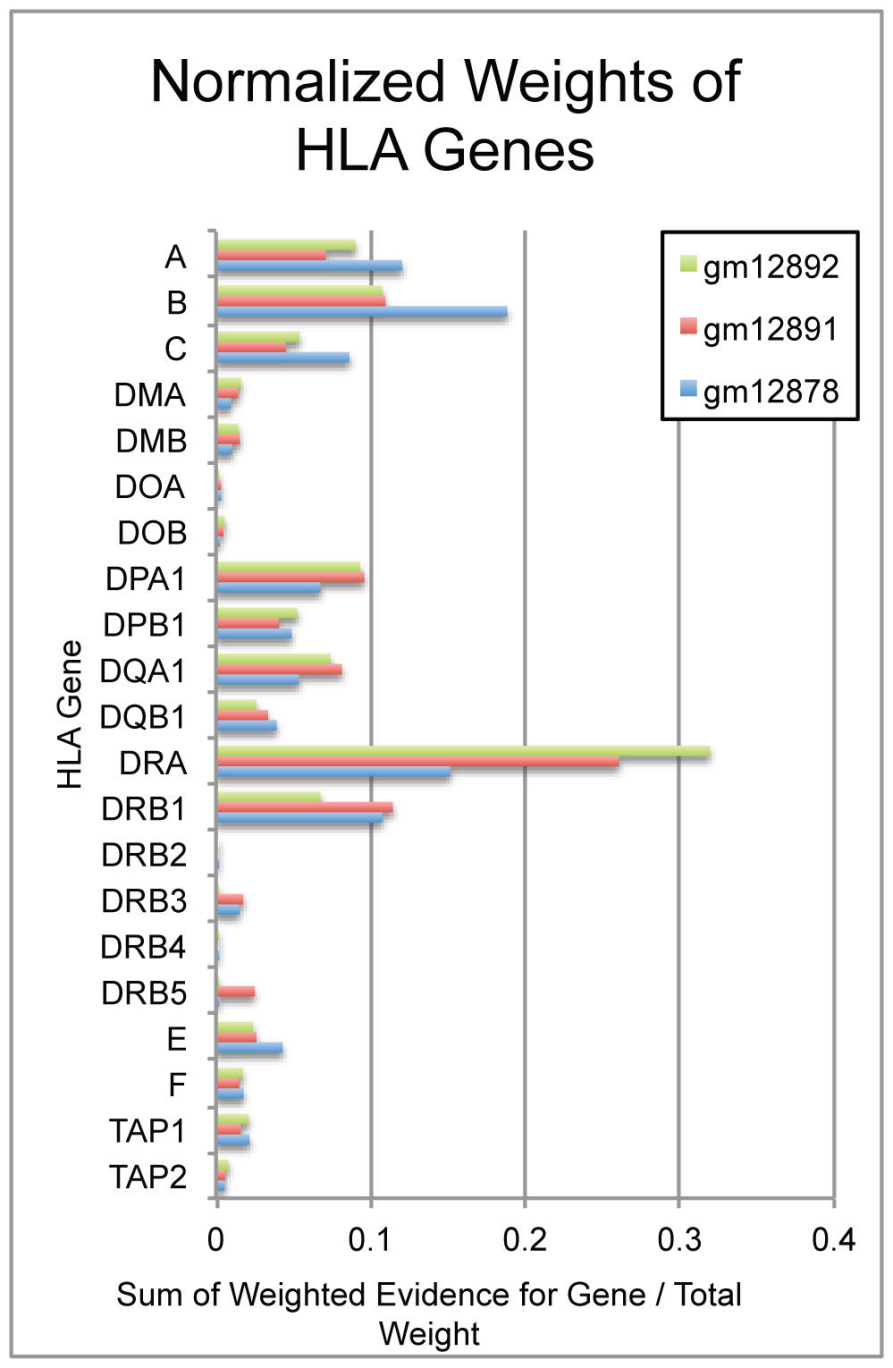
Final pruned weights supporting each gene in the IMGT database shows expression over major class I molecules (A, B, C) as well as over most major class I molecules (DMA, DMB, DPA1, DPB1, DQB1, DRA, DRB1). Some expression is seen in minor class I alleles (E, F) and non classical molecules (TAP1 and TAP2).

In addition to predicting haplotypes, the method allows for the estimation of expression of all genes. Figure 5 shows the number of pruned reads aligning to each HLA gene. We see that classical class I molecules (*HLA-A*, *HLA-B*, *HLA-C*) have moderate expression in all samples. Likewise there is moderate to high expression of some major class II genes (*HLA-DPA1*, *HLA-DPB1*, *HLA-DQA1*, *HLA-DQB1*, *HLA-DRA* and *HLA-DRB1*). There is lower expression of the minor class I molecules along with some class II molecules (*HLA-E*, *HLA-F*, *HLA-DMA*, *HLA-DMB*, *HLA-DOA* and *HLA-DOB*).

### HapMap Results

Fifty HapMap samples that have been both HLA haplotyped with Sanger Sequencing and for which there are RNA-seq data available were analyzed using HLAforest. RNAseq data was 2x37nt in length. HLAforest was able to predict 96% of allele-group level haplotypes correctly. 82% of peptide-level haplotypes were also predicted correctly. When alignments were restricted to disallow mismatches, HLAforest was able to predict 96.5% and 83.5% of peptide level haplotypes correctly (Table 2). On average, 127,000 reads aligned to the IMGT database when mismatches were allowed and 91,000 reads aligned when no mismatches were allowed. Detailed results are available in Table S4.

**Table 2:** Accuracy of haplotyping results from 50 HapMap samples with 2x37bp reads allowing or not allowing mismatched alignments to references.

	**Mismatches Allowed?**	**A**	**B**	**C**	**DQA**	**DQB**	**DRB**	**Class I**	**Class II**	**Total**
**Allele Group**	Yes	.950	.930	.950	.980	.980	.976	.943	.978	.960
**Peptide**	Yes	.940	.838	.680	.804	.804	.872	.819	.823	.821
**Allele Group**	No	.950	.940	.950	1	.990	.963	.947	.985	.965
**Peptide**	No	.940	.848	.730	.804	.825	.872	.839	.830	.835

### Colorectal Cancer Samples

In a study by Warren, et al. on HLA haplotyping using short read assembly, he presented a dataset of sixteen RNA-seq samples for which Class I molecules were haplotyped by PCR [22]. Of the 96 total possible alleles, 87 were typed to the peptide level. Reads for this dataset were 2x100nt in length. On average, 3,500 reads aligned to the IMGT database. HLAforest predicted 85 (97.7%) of low resolution haplotypes correctly and 74 (85%) of high resolution peptides correctly. Detailed results are available in Table S5.

## Discussion

The method described herein performed well in simulations with read length and substitution rates mirroring those of available sequencing technologies, namely Illumina sequencers. HLAforest has the advantage of scaling well with longer reads and it fully utilizes the phasing information present in paired-end reads. The method is generalizable to any set of genes that can be arranged hierarchically. It also has the major benefit of selecting haplotypes individually, thus reducing the complexity and combinatorial difficulty of selecting two haplotypes simultaneously. Some problems remain, including the inability to call novel haplotypes and report ambiguous haplotypes, but these can be addressed in the future.

Simulations show that this method can achieve a high resolution accuracy of 93% (2x100bp reads and 0.5% substitution rate) over the genes that represent the majority of diversity in the IMGT database (*HLA-A*, *HLA-B*, *HLA-C*, *HLA-DRB1*). Evaluation of this method on the daughter–father-mother cell line trio shows that 26/30 (87%) the daughter’s alleles were predicted consistently at the peptide level. After comparison to results of Sanger sequencing and targeted resequencing with Roche 454, it was determined that errors in typing occurred within gm12891 and gm12892 (Table 1). Errors in typing may be related to the 2x75bp read lengths available for these cell lines. In order to make accurate predictions, HLAforest relies on the phasing information within individual reads, which are dependent on read length.

The majority of information extracted from short reads comes from the ability to phase discriminatory SNPs across the most diverse coding regions of the HLA genes. For class I genes, the majority of diversity are present in exons 2-4 [31]. Here, short reads of sufficient length and quality are able to phase the discriminatory SNPs that define each haplotype. Indeed, increasing the read length to 2x200 nt in simulations substantially improves the accuracy of the method with accuracy greater than 96.7% at the peptide level. This finding is significant as high throughput sequencing technologies such as Illumina and Ion Torrent have announced plans to release 2x200 nt sequencing kits. The HLAforest method is best applied to RNA-seq data as reads are more likely to phase discriminatory SNPs across exons in fully spliced transcripts. If read lengths or insert size exceed the length of introns in these genes, this method can be extended to the whole genome or targeted sequencing datasets without loss of accuracy.

The robustness of this method is apparent in simulations testing effect of substitution rate on accuracy. Modern Illumina machines report an overall substitution rate < 0.5% [32]. In simulations with comparable error rates, HLAforest is able to predict 93% of haplotypes at the peptide level. Even with substitution rates as high as 8.8%, HLAforest can predict 99% of low resolution haplotypes correctly.

This method has major benefits over earlier methods. First it can be generalized to any set of genes that are classified hierarchically. Secondly, as opposed to competing methods, there are no combinatorial issues with selecting two haplotypes to score simultaneously. In this method, haplotypes are chosen procedurally and this reduces the computation time necessary for scoring many hypothetical pairs of alleles.

Recently, HLAMiner has been published and shares many of the same benefits as HLAforest. The major distinction between the methods is HLAMiner’s reliance on the *de novo* assembler, TASR [33]. HLAforest exploits highly efficient short read alignment algorithms, which have been the subject of major development in the bioinformatics field. This alignment step is easily parallelizable, as opposed to *de novo* assembly methods that require shared memory. Additionally, HLAforest uses all the phasing information within paired-end reads rather than attempting *de novo* assembly with shorter k-mers. Differences in the methodologies make direct comparison of the methods difficult. However, our simulations with 2x100 nt reads and 0.55% substitution rate show an average major class I accuracy of 92.7% at the peptide level as opposed to HLAMiner’s sensitivity and specificity of 84.7% and 89.65% with the same parameters, respectively. When error rates were increased to 2%, average major class I accuracy with HLAforest dropped slightly to 92.7% whereas the sensitivity and specificity of HLAminer’s simulations were 54.9% and 87.5% respectively. The data suggests that HLAforest’s predictions are more accurate than HLAMiner even with larger error rates.

When compared to HLAMiner on sixteen colorectal cancer samples, HLAforest was able to predict 97.7% of low resolution haplotypes and 85% of high resolution haplotypes for Class I molecules. HLAMiner reported 95.6% sensitivity and 99% specificity at low resolution, and 90.7% sensitivity and 93.5% specific at high resolution. It is worth noting that the sensitivity and specificity measures reported by Warren, et al. are not standard. Whereas HLAforest generated 96 predictions for the 96 possible peptide-level Class I haplotypes, HLAMiner generated 235 predictions.

HLAforest’s predictions for colorectal cancer samples fell below the levels predicted by simulations with 2x100nt read lengths. This is perhaps due to the low number of reads aligning to the IMGT database, especially when compared to the HapMap samples (3,500 for colorectal samples vs 127,000 for HapMap samples).

HLAforest performed well on fifty HapMap samples using 2x37nt reads. It was able to predict 96% low resolution haplotypes accurately. Performance of HLAforest was comparable to seq2HLA, which predicted low resolution haplotypes with 100% sensitivity and 93% specificity. However, HLAforest was also able to predict 82% of high resolution haplotypes correctly. Remarkably, HLAforest performed better on these samples than it did in simulation with similar read lengths. This is perhaps due to the increased coverage in the HapMap samples as well as the reduced representation of haplotypes present in these samples. These cross study comparisons imply better performance with HLAforest; however, such comparisons should be interpreted cautiously until systematic benchmarking can be performed.

Although HLAforest presents many strengths, there are some shortcomings. First, typing class II MHC molecules may be impossible based on the cell type of the input RNA. Because only specialized antigen presenting cells (such as B cells, dendritic cells, etc) express class II MHC molecules, some of these cells must be present in the sample in order to generate the corresponding reads. Second, the method is restricted to haplotypes that already exist in the IMGT database. HLAforest chooses the closest matching haplotype, but does not reconstruct the actual haplotype. It is feasible to look for novel haplotypes by generating a consensus sequence from the reads supporting each predicted haplotype and then checking for novel SNPs. Third, HLAforest reports only two haplotypes and does not produce confidence scores at this time. Finally, this method does not yet incorporate population-based frequency data that has been shown to improve the accuracy of all typing methods in well-studied populations [34]

## Supporting Information

Figure S1 **Number of Haplotypes in IMGT Database.**
(PNG)

Table S1 **IMGT Statistics.**
(XLS)

Table S2 **Simulation Conditions and Results.**
(XLS)

Table S3 **Cell Line Trio Results.**
(XLS)

Table S4 **HapMap Results.**
(XLS)

Table S5 **Colorectal Cancer Results.**
(XLS)
