# Supplementary figures and images for: HLA Haplotyping from RNA-seq Data Using Hierarchical Read Weighting
Source: PLoS One. 2013 Jun 28;8(6):e67885. doi: 10.1371/journal.pone.0067885 (PMC3696101; doi:10.1371/journal.pone.0067885)

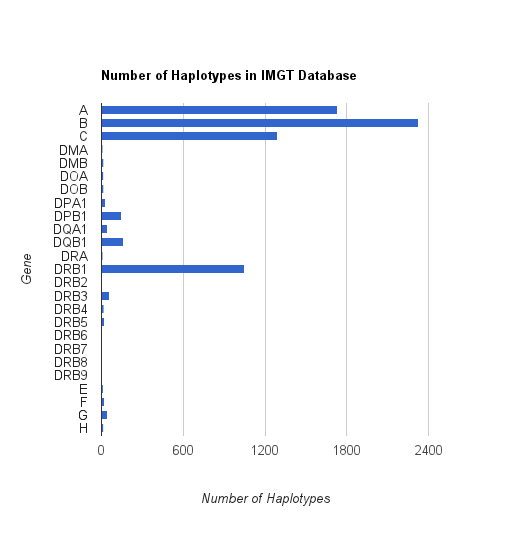

Supplement: Figure S1 — Number of Haplotypes in IMGT Database. (PNG) [file pone.0067885.s001.png]
